# Identification of a lncRNA AC011511.5- Mediated Competitive Endogenous RNA Network Involved in the Pathogenesis of Allergic Rhinitis

**DOI:** 10.3389/fgene.2022.811679

**Published:** 2022-05-31

**Authors:** Yujuan Yang, Qi Sun, Jing Guo, Zhen Liu, Jianwei Wang, Yao Yao, Pengyi Yu, Jiayu Cao, Yu Zhang, Xicheng Song

**Affiliations:** ^1^ Department of Otorhinolaryngology, Head and Neck Surgery, Yantai Yuhuangding Hospital, Qingdao University, Yantai, China; ^2^ Shandong Provincial Clinical Research Center for Otorhinolaryngologic Diseases, Yantai, China

**Keywords:** allergic rhinitis, lncRNA, ceRNA network, miRNA, lncRNA AC011511.5

## Abstract

LncRNA-miRNA-mRNA competing endogenous RNA (ceRNA) networks are thought to be involved in regulating the development of various inflammatory diseases. Up to now, the mechanism of such a network in allergic rhinitis (AR) remains unclear. In the study, we investigated the differential expression of lncRNAs (DElncRNAs) and mRNAs (DEmRNAs) by performing a microarray analysis of peripheral blood obtained from AR patients and healthy control subjects. StarBase 2.0 was used to predict miRNAs that might interact with various DElncRNAs and DEmRNAs. We constructed a ceRNA network based on potential lncRNA-miRNA-mRNA interactions. The Cluster Profiler R package was used to perform a functional enrichment analysis of the hub-ceRNA, and Molecular Complex Detection (MCODE) was used for further identification of the hub-ceRNA network. The expression levels of genes contained in the hub-ceRNA network were validated by RT-PCR. In total, 247 DEmRNAs and 18 DelncRNAs were aberrantly expressed in the PBMCs of AR patients. A ceRNA network consisting of 3 lncRNAs, 45 miRNAs, and 75 mRNAs was constructed. A GO analysis showed that negative regulation of immune response, response to interferon-beta, and response to interferon-alpha were important terms. A KEGG pathway analysis showed that 75 mRNAs were significantly enriched in “NOD-like receptor signaling pathway” and “tryptophan metabolism”. Ultimately, a hub-ceRNA network was constructed based on 1 lncRNA (AC011511.5), 5 miRNAs (hsa-miR-576-5p, hsa-miR-520c-5p, hsa-miR-519b-5p, hsa-miR-519c-5p, and hsa-miR-518d-5p), and 2 mRNAs (ZFP36L1 and SNX27). Following further verification, we found that overexpression of lncRNA AC011511.5 or inhibitor of miR-576-5p upregulated SNX27 expression. The expression of SNX27 in the lncRNA AC011511.5 overexpression & miR-576-5p inhibitor group was not different from that in the miR-576-5p inhibitor group or lncRNA AC011511.5 overexpression group, indicating that overexpression of lncRNA AC011511.5 could not further upregulate the expression of SNX27 in miR-576-5p inhibitor Jurkat cells. This network may provide new insights to search for biomarkers that can be used for the diagnosis and clinical treatment of AR.

## Introduction

Allergic rhinitis (AR) characterized by symptoms of congestion, runny nose, sneezing and itching, is emerging as a global health problem (Meng et al., 2019; Zhang et al., 2021). It is estimated that the global prevalence rate of AR is as high as 10%–30%. About 40% of patients with AR are susceptible to asthma, which seriously affects the quality of life of patients and brings a huge economic burden to society (Brożek et al., 2017). Like other allergic diseases, the pathogenesis of AR is complex and unclear. This lack of clarity can make it difficult to treat AR. Therefore, there remains an urgent need to identify key pathogenic molecules involved in AR pathologies, as discovery of those molecules may help investigators better understand the underlying molecular mechanisms of AR and identify new therapeutic targets for AR.

The term long non-coding RNA (lncRNA) refers to a class of RNAs that contain >200 nucleotides, lack an open reading frame, and are involved in regulating gene transcription and post-transcription activities (Marchese et al., 2014; Engreitz et al., 2016; Quinn and Chang, 2016). Numerous studies suggest that lncRNAs play an important role in the pathogenesis of various diseases, including AR (Zhu et al., 2020; Zhou et al., 2021). Our previous research found that certain lncRNAs are differentially expressed in patients with AR (Yang et al., 2020). A growing body of evidence indicates that lncRNAs can act as competitive endogenous RNAs (ceRNAs) that bind to miRNAs via their miRNA response elements, and thereby affect and regulate the expression of target genes (Salmena et al., 2011; Wu et al., 2020). MicroRNAs (miRNAs) inhibit the expression of target genes by binding to complementary sequences in the 3′-untranslated regions (UTRs) of their target mRNAs (Hausser and Zavolan, 2014). In recent years, miRNAs have been widely used for identifying candidate genes and exploring the mechanisms of lncRNAs by constructing ceRNA-regulated networks based on a bioinformatics analysis. These networks may provide a variety of clues to illuminate the pathogenesis of diseases. However, few reports have described how to construct a lncRNA-related ceRNA network and identify a key lncRNA-miRNA-mRNA axis involved in the pathogenesis of AR.

**FIGURE 1:**
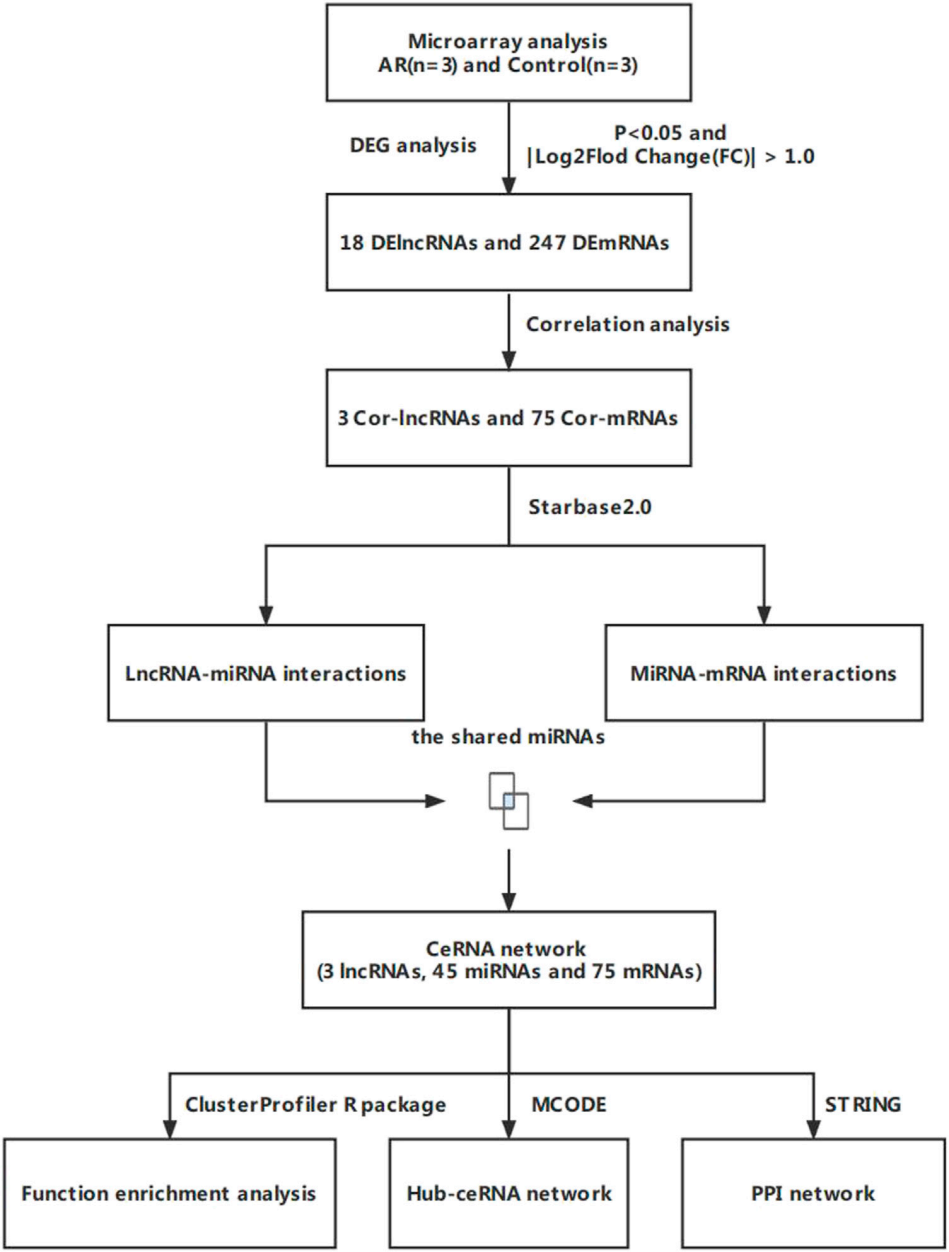
Flow chart for the ceRNA network analysis.

In the study, we used microarray analysis to explore differentially expressed lncRNAs in patients with AR. We also used public databases to construct a lncRNA-related ceRNA regulatory network for the purpose of identifying the regulatory mechanism of lncRNA-miRNA-mRNA mediated ceRNA in the pathogenesis of AR (Figure 1).

## Materials and Methods

### Microarray Data Information

In our previous study, 3 patients with AR (2 males and 1 female; mean age 30.0 years), which were diagnosed according to Chinese Society of Allergy Guidelines for Diagnosis and Treatment of Allergic Rhinitis (Cheng et al., 2018) and 3 control subjects (2 males and 1 female; mean age 30.7 years) were enrolled. All patients had positive allergen-specific IgE, had not received immunotherapy during the previous 3 months, and had no other allergic diseases. We excluded patients who smoked and who had systemic immune or infectious diseases. All control subjects have negative allergen-specific IgE. The patients and control subjects provided their signed informed consent. The total RNA was extracted from whole blood (8 ml) by using a Total RNA Extraction Micro Kit (RNT411, Mabio, Guangzhou, China) and the RNA quality was evaluated using NanoDrop 2000 software. The RNA was reverse transcribed into complementary deoxyribose nucleic acid (cDNA) by using a Total-Transcriptome cDNA Synthesis Kit (Applied Biological Materials Inc., Canada). CapitalBio Technology Human LncRNA Array V4, 4 × 180 K chip which was performed by Beijing Capitalbio Technology Co., Ltd., China was used to detect Human gene expression profile and the test method was numbered as AG-GE-WL10-01-20 (Yang et al., 2020).

In this study, the limma R package was used for differential analysis, and transcripts with a *p*-value < 0.05 and |Log2Fc| > 1.0 were used to distinguish the differentially expressed mRNAs (DEmRNAs) and differentially expressed lncRNAs (DElncRNAs). According to the mechanisms of ceRNA, the expression of mRNAs and lncRNAs have the same orientation relationship, the expression of lncRNAs might be positively correlated with their regulated mRNAs (Chu et al., 2020). The co-expressed lncRNAs and co-expressed mRNAs were identified by using the Pearson correlation analysis (coefficient >0.9 and *p* < 0.001) among the DEmRNAs and DElncRNA and were included in the follow-up analysis.

### Construction of a lncRNA-miRNA-mRNA Mediated ceRNA Network in AR

Due to the lack of a miRNA expression profile for patients with AR, we predicted the miRNAs by using Starbase2.0 (http://starbase.sysu.edu.cn/). Starbase2.0 could provide comprehensive prediction of lncRNA-mRNA interactions and miRNA-mRNA interactions (Li et al., 2014). The overlapping miRNAs between lncRNA-miRNA interactions and mRNA-miRNA interactions were included in a follow-up analysis as candidate miRNAs of the ceRNA network, and it was visualized by Cytoscape software (Version 3.8.0, http://cytoscape.org).

### Functional Enrichment Analysis of DEmRNAs in the ceRNA Network

In order to reveal the function of the ceRNA network, ClusterProfiler R package (https://bioconductor.org/packages/release/bioc/html/clusterProfiler.html, the significant selection criteria were *p* < 0.05) was used for functional enrichment analysis of mRNA in the ceRNA network, including gene ontology (GO) and Kyoto Encyclopedia of Genes and Genomes (KEGG). The enrichment results were visualized using bioinformatics online website (http://www.bioinformatics.com.cn/).

The indicated interactions between the relevant mRNAs were made more intuitive by creating a protein interaction network (PPI network) by using the string database (http://string-db.org/) (minimum required interaction score = 0.400 and hide disconnected nodes in the network) to explore the core genes. The PPI network was constructed using the Cytoscape software. The modules interactions in the PPI network were identified using the cytoscape MCODE module (Molecular Complex Detection, degree cutoff = 2, node score cutoff = 0.2, k-core = 2, and max. Depth = 100) and the highly interconnected modules were identified as the hub-mRNAs.

### Filtering of Hub ceRNA Network

The cytoscape MCODE module (degree cutoff = 2, node score cutoff = 0.2, k-core = 2, and max. Depth = 100) which was a plug-in for discovering closely connected hub-ceRNA networks existing within complex networks based on topology was used to discover the most important clusters in the ceRNA network, and the highest score cluster was found to be hub-ceRNA.

### Peripheral Blood Mononuclear Cell Isolation

In this study, a total of 21 AR patients (13 males and 8 females; mean age 27.9 years) and 16 control subjects (8 males and 8 females; mean age 37.1 years) peripheral blood were collected for real-time fluorescence quantitative polymerase chain Reaction (RT-PCR) analysis. Diagnosis and exclusion criteria were the same as the screening cohort. The patients and control subjects provided their signed informed consent. The study protocol was approved by the ethics committee of Yantai Yuhuangding Affiliated Hospital of Qingdao University (Approval NO. 2020-295).

8 ml of venous blood was collected from each subject and then was centrifuged at 2000 rpm for 10 min, then the plasma was removed. The precipitate was treated with phosphate buffered saline (1:1) and 4 ml Ficoll was added. After centrifugation, 4 ml of red blood cell lysate was added to the white membranous layer and the tube was centrifuged at 1,600 rpm for 20 min.

### Cell Culture and Gene Expression Interference

Jurkat cells were cultured in RPMI 1640 medium supplemented with 10% fetal bovine serum and 1% penicillin/streptomycin at 37°C with 5% CO_2_. Briefly, 2 × 10^6^ Jurkat cells were plated in a 12-well plate. LncRNA AC011511.5 overexpressing lentivirus (constructed by Shanghai Genechem Co., LTD., MOI = 20) was added for transfection. The Jurkat cells were transfected with miR-576-5p inhibitor contained in lentivirus (constructed by Gene Pharma, MOI = 20) to obtain miR-576-5p inhibitor cells. After 72 h of transfection, the cells were then incubated with 1 μg/ml puromycin. All products were compared to those produced by the corresponding negative control.

### RNA Extraction and RT-PCR Verification

The total RNA was extracted from PBMCs by using SparkZol Reagent (SparkJade, Shandong, China) and then reverse transcribed into cDNA by using an Evo M-MLV RT Kit containing gDNA Clean for qPCR II (Accurate Biology, (Hunan), Co., Ltd.). The expression levels of lncRNAs and mRNAs were detected by RT-PCR, which was performed by using a SYBR Green qPCR Mix kit (With ROX) (SparkJade, Shandong, China). The conditions used for qPCR were as follows: 95°C for 2 min, followed by 40 cycles of 95°C for 10 s, 60°C for 30 s. The total RNA was reverse transcribed into miRcDNA by using a miRNA first Strand cDNA Synthesis Kit (by stem-loop) (Vazyme, Nanjing, China). miRNA expression was achieved by performing RT-PCR with miRNA universal SYBR qPCR Master Mix (Vazyme, Nanjing, China) under the following conditions: 95°C for 5 min, followed by 40 cycles of 95°C for 10 s and 60°C for 30 s. The primers used for amplification of lncRNAs, miRNAs, and mRNAs are shown in Table 1. Each RT-qPCR analysis was repeated more than three times. Relative levels of gene expression were calculated by using the 2^−ΔΔCT^ method (Ayuk et al., 2016).

**TABLE 1:** Primer sequences used for RT-qPCR.

Gene	Primer Sequence
*GAPDH*	Forward: TGA​CTT​CAA​CAG​CGA​CAC​CCA
	Reverse: CAC​CCT​GTT​GCT​GTA​GCC​AAA
*U6*	Forward: CTCGCTTCGGCAGCACA
	Reverse: AAC​GCT​TCA​CGA​ATT​TGC​GT
*lncRNA AC011511.5*	Forward: TAG​TGC​AAG​CTC​CCA​GTG​AA
	Reverse: CCT​CCC​CAC​CCA​CAT​ACA​TT
*Hsa-miR-576-5p*	Forward: GCG​CGA​TTC​TAA​TTT​CTC​CAC
	Reverse: AGT​GCA​GGG​TCC​GAG​GTA​TT
*Hsa-miR-518d-5p*	Forward: CGC​GCT​CTA​GAG​GGA​AGC​AC
	Reverse: AGT​GCA​GGG​TCC​GAG​GTA​TT
*Hsa-miR-519b/c-5p*	Forward: CGCTCTAGAGGGAAGCGC
	Reverse: AGT​GCA​GGG​TCC​GAG​GTA​TT
*Hsa-miR-520c-5p*	Forward: CGC​GCT​CTA​GAG​GGA​AGC​AC
	Reverse: AGT​GCA​GGG​TCC​GAG​GTA​TT
*SNX27*	Forward: CAA​GTC​CGA​GTC​CGG​CTA​C
	Reverse: CCT​GCT​CGA​ATC​AGG​TCC​A
*ZFP36L1*	Forward: ACT​CCA​GCC​GCT​ACA​AGA​C
	Reverse: CGT​AGG​GGC​AAA​AGC​CGA​T

### Statistical Analysis

All statistical analyses were performed by using GraphPad Prism 7.0 (Graphpad Software, Inc., La Jolla, CA, United States). Results for continuous variables were expressed as a mean value ±standard deviation. The significance of differences between groups was evaluated by using the student’s t test or Fisher’s exact test. A *p*-value < 0.05 was considered statistically significant.

## Results

### 247 DEmRNAs and 18 DElncRNAs Were Identified in AR by Microarray Detection

**FIGURE 2:**
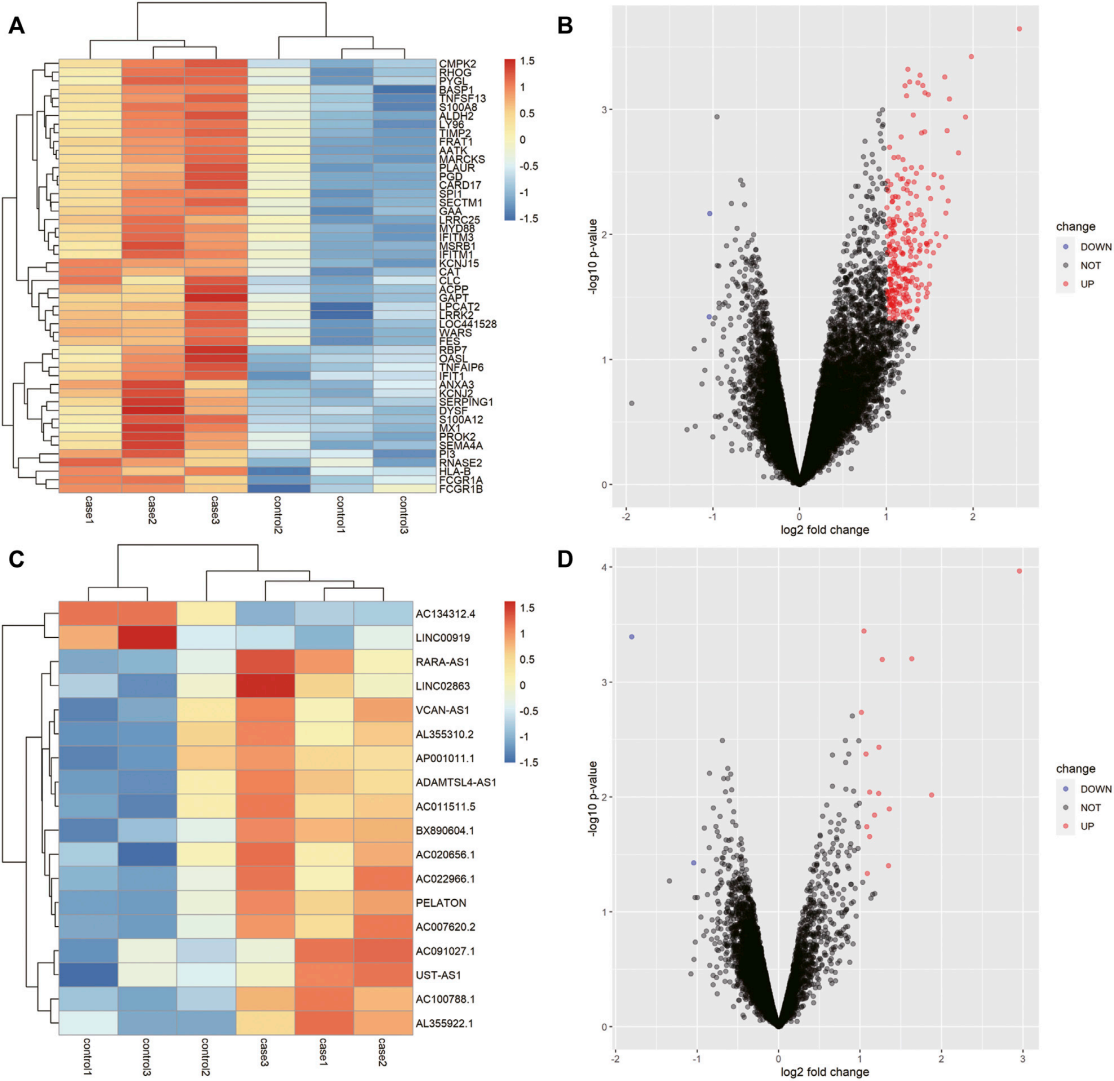
LncRNAs and mRNAs that were differentially expressed in the AR patients. **(A)** Heat map of the first 50 differentially expressed mRNAs in AR. **(B)** Volcano map of the first 50 differentially expressed mRNAs in AR. **(C)** Heat map of the 18 differentially expressed lncRNAs in AR. **(D)** Volcano map of the 18 differentially expressed lncRNAs in AR.

Based on the chip sequencing data, we identified 20844 mRNAs and 12177 lncRNAs. Totals of 247 DEmRNAs (245 up-regulated and 2 down-regulated) and 18 DElncRNAs (16 up-regulated and 2 down-regulated) were screened by difference analysis. The top50 DEmRNAs and all DElncRNAs were visualized by using a Volcano plot and heat map (Figure 2). Based on a correlation analysis, 3 co-expressed lncRNAs (lncRNA AC007620.2, lncRNA BX890604.1 and lncRNA AC011511.5) and 75 co-expressed mRNAs (Supplementary Table S1) were selected for follow-up analysis.

### Construction of the lncRNA-Associated ceRNA Network in AR

**FIGURE 3:**
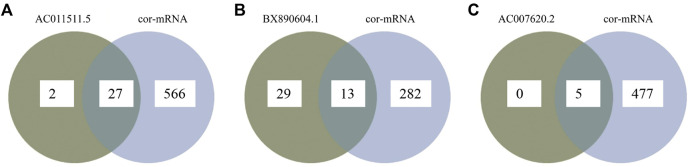
Overlapping between lncRNA-related miRNAs and mRNA-related miRNAs as predicted miRNAs by Starbase2.0. **(A)** Overlapping miRNAs between lncRNA AC007620.2-related miRNAs and its co-expressed mRNA-related miRNAs; **(B)** Overlapping miRNAs between lncRNA BX890604.1-related miRNAs and its co-expressed mRNA-related miRNAs; **(C)** Overlapping miRNAs between lncRNA AC011511.5-related miRNAs and its co-expressed mRNA-related miRNAs.

**FIGURE 4:**
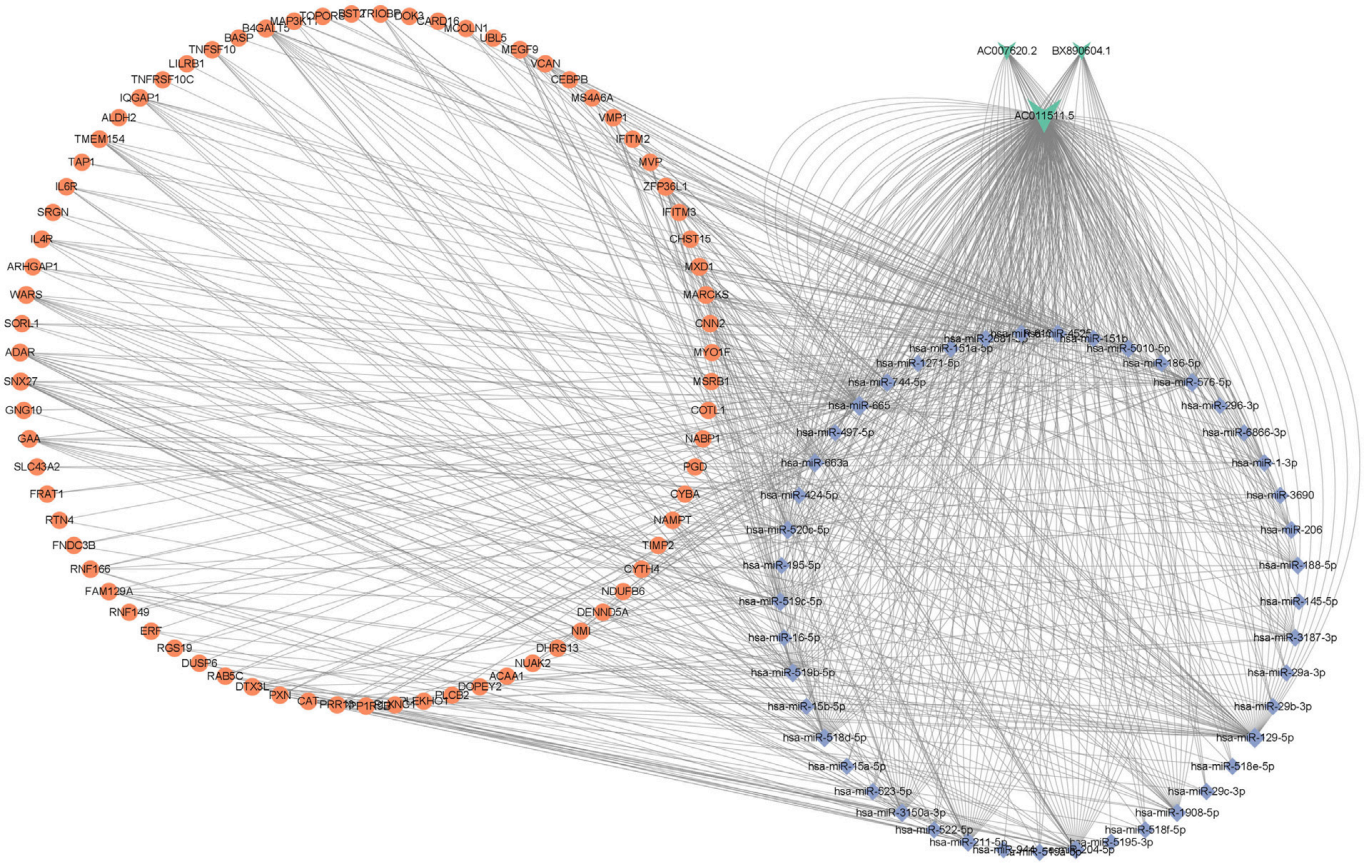
The ceRNA network of lncRNA-miRNA-mRNA in AR, including 3 lncRNAs, 45 miRNAs, and 75 mRNAs. V-shapes represent lncRNAs, diamonds indicate miRNAs, and rounds indicate mRNAs.

ceRNA crosstalk was identified by the miRNAs shared between lncRNAs and mRNAs. There were 5 intersecting miRNAs between lncRNA AC007620.2 and its co-expressed mRNAs, 13 intersecting miRNAs between lncRNA BX890604.1 and its co-expressed mRNAs and 27 intersecting miRNAs between lncRNA AC011511.5 and its co-expressed mRNA. Finally, a total of 45 lncRNA-miRNA-mRNA interactions (Figure 3 and Supplementary Table S2) were identified by Starbase2.0. A ceRNA network (Figure 4 and Supplementary Table S3) consisting of 712 edges and 123 nodes (including 3 lncRNAs, 45 miRNAs, and 75 mRNAs) was constructed. These RNA interactions may play a key role in the pathogenesis of AR.

### mRNAs in the ceRNA Network Were Enriched in Immune Response

**FIGURE 5:**
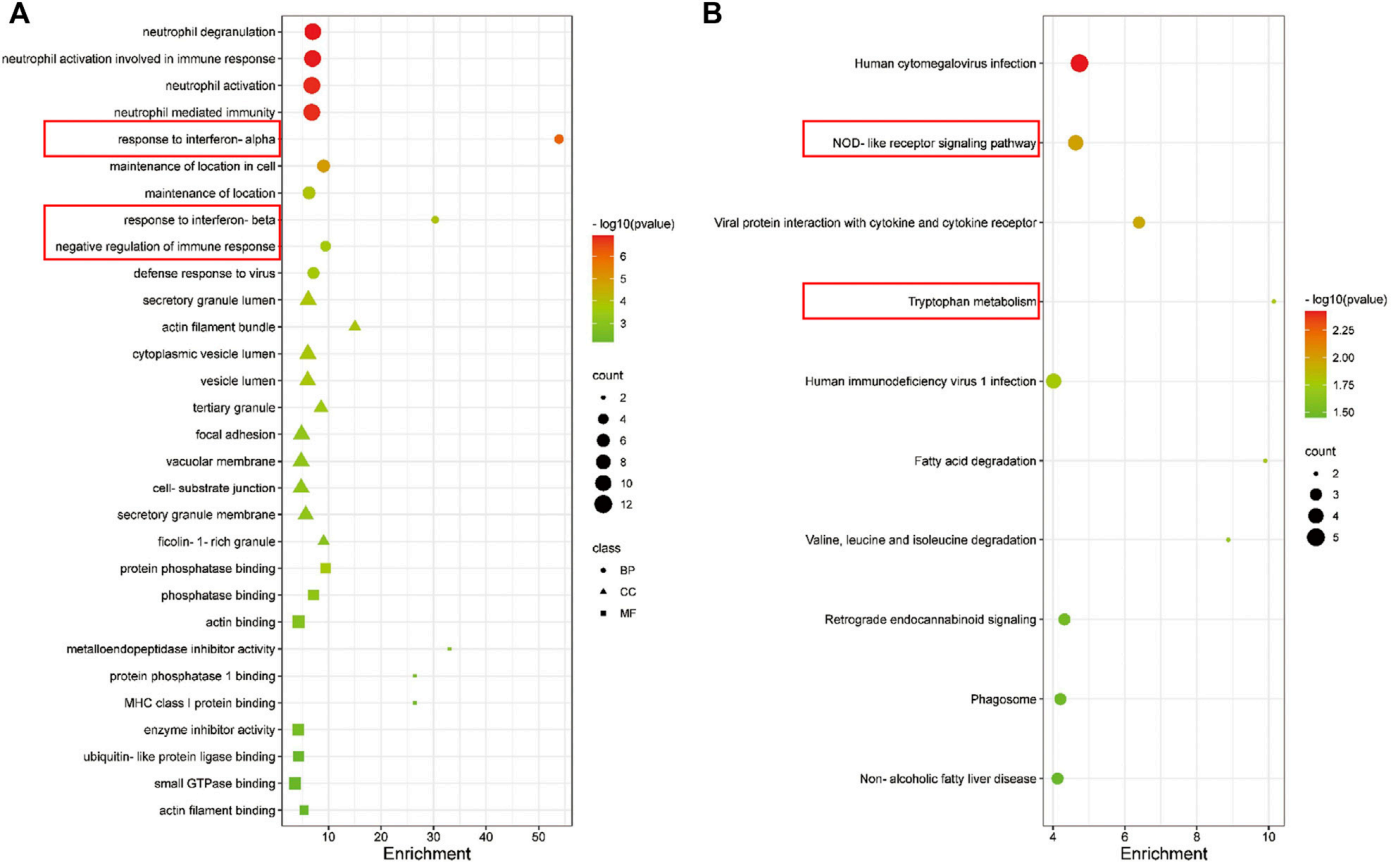
GO and KEGG enrichment analyses of mRNAs in the ceRNA in AR patients. **(A)** GO enrichment analysis of mRNAs. **(B)** The first 10 pathways in the KEGG enrichment analysis of mRNAs.

**FIGURE 6:**
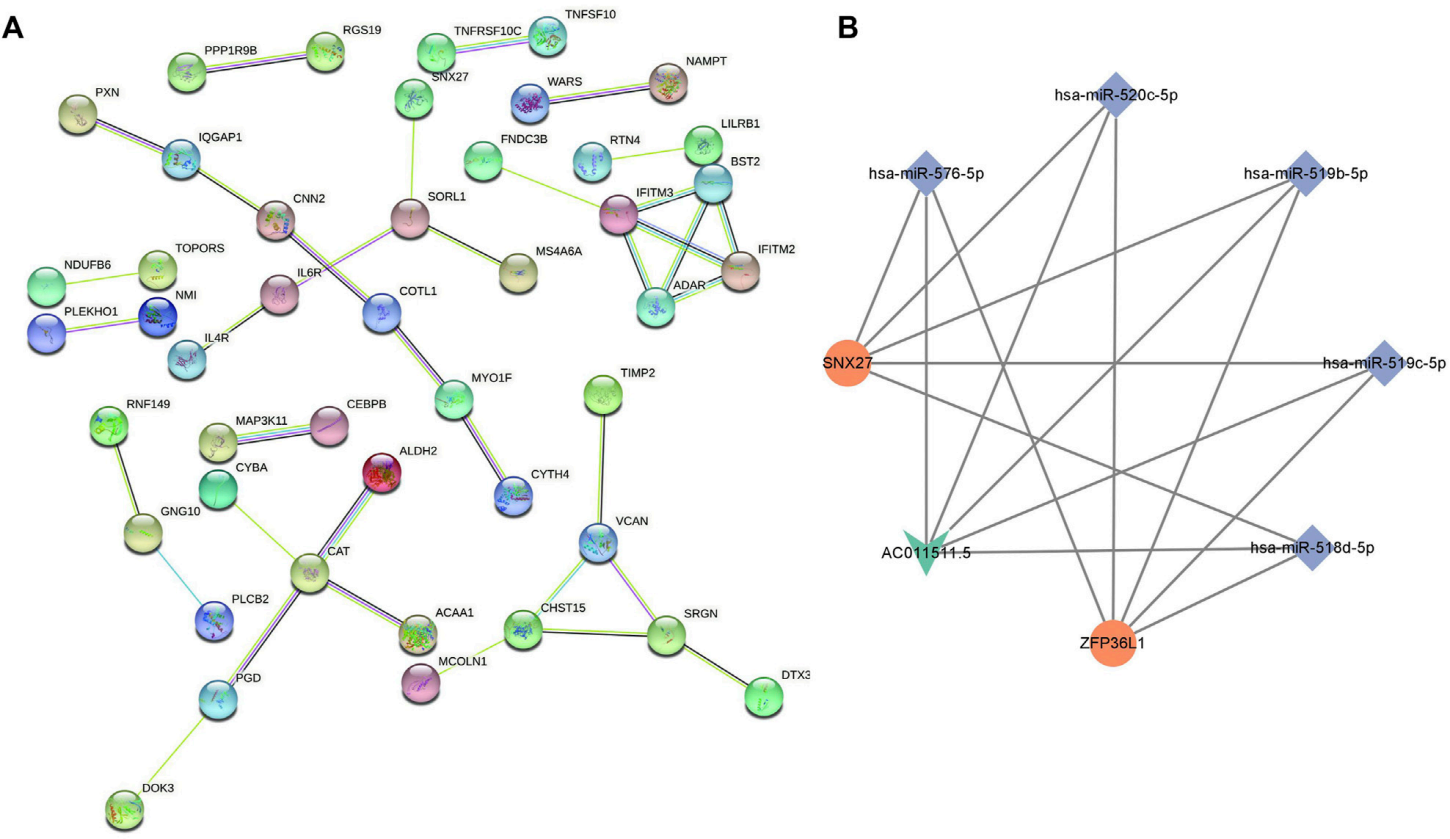
PPI network of mRNAs and the lncRNA-miRNA-mRNA subnetwork in AR. **(A)** PPI network of mRNAs. **(B)** lncRNA-miRNA-mRNA subnetwork in AR: V-shapes represent lncRNAs, diamonds indicate miRNAs, and rounds indicate mRNAs.

The biological function of the ceRNA network was explained by a GO analysis and KEGG analysis. In biological processes, the mRNAs were mainly involved in negative regulation of immune response, response to interferon-beta, response to interferon-alpha, and neutrophil mediated immunity. In terms of cellular components, the mRNAs were mainly enriched in ficolin-1-rich granule, cell-substrate junction, and vacuolar membrane. Actin filament binding, small GTPase binding, ubiquitin-like protein ligase binding, and enzyme inhibitor activity are mainly related to molecular function (Figure 5A). As shown in the figure, a KEGG pathway analysis revealed that 75 mRNAs in the AR blood samples were significantly enriched in viral protein interaction with cytokine and cytokine receptor; valine, leucine and isoleucine degradation, tryptophan metabolism, retrograde endocannabinoid signaling, phagosome, non-alcoholic fatty liver disease, NOD-like receptor signaling pathway, human immunodeficiency virus 1 infection, human cytomegalovirus infection, and fatty acid degradation (Figure 5B). The PPI network consisting of 36 edges and 45 nodes was established for indicating the interaction among the determined mRNAs in the ceRNA network more intuitively (Figure 6A). The highly interconnected modules in the PPI network were identified as the hub-mRNAs and 4 genes were gained, including IFITM2, IFITM3, BST2 and ADAR.

### Construction of the Hub ceRNA Network in AR

We used the cytoscape MCODE module to discover the most important cluster in the ceRNA network, and the highest score cluster was found to be hub-ceRNA. Finally, we constructed a hub-ceRNA network composed of 1 lncRNA (AC011511.5), 5 miRNAs (hsa-miR-576-5p, hsa-miR-520c-5p, hsa-miR-519b-5p, hsa-miR-519c-5p, and hsa-miR-518d-5p), and 2 mRNAs (the zinc finger protein 36-like 1, ZFP36L1 and SNX27) based on the above ceRNA network (Figure 6B).

### Verification of the Hub ceRNA Network by RT-PCR

**FIGURE 7:**
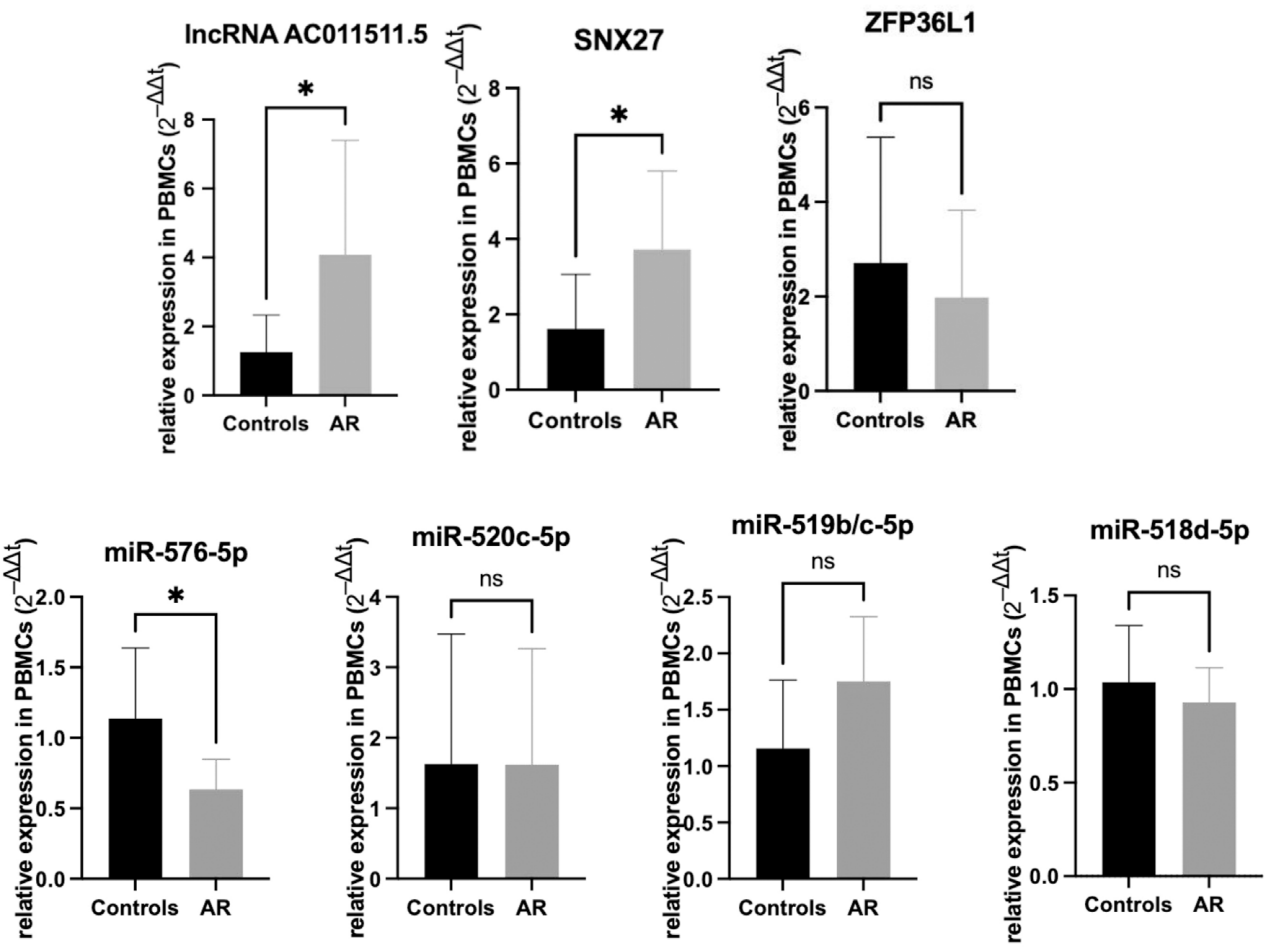
Expression verification of lncRNA AC011511.5, miRNAs, and mRNAs in the hub-network in the peripheral blood mononuclear cells from AR patients and the control group. *p < 0.05.

We detected the expression levels of lncRNAs, miRNAs and mRNAs involed in hub-ceRNA in the AR patients and control subjects. The results showed that lncRNA AC011511.5 (*p* = 0.037) and SNX27 (*p* = 0.020) were more highly expressed in the AR patients than that in the control subjects, while has-miR-576-5p (*p* = 0.0137) was expressed at significantly lower levels in the AR patients than in the control subjects. There was no significant difference in the expression levels of ZFP36L1 (*p* = 0.445), hsa-miR-520c-5p (*p* = 0.991), hsa-miR-519b/c-5p (*p* = 0.095), and hsa-miR-518d-5p (*p* = 0.434) in the AR patients and control subjects (Figure 7).

### LncRNA AC011511.5 Regulates the Expression of SNX27 *via* miR-576-5p

**FIGURE 8:**
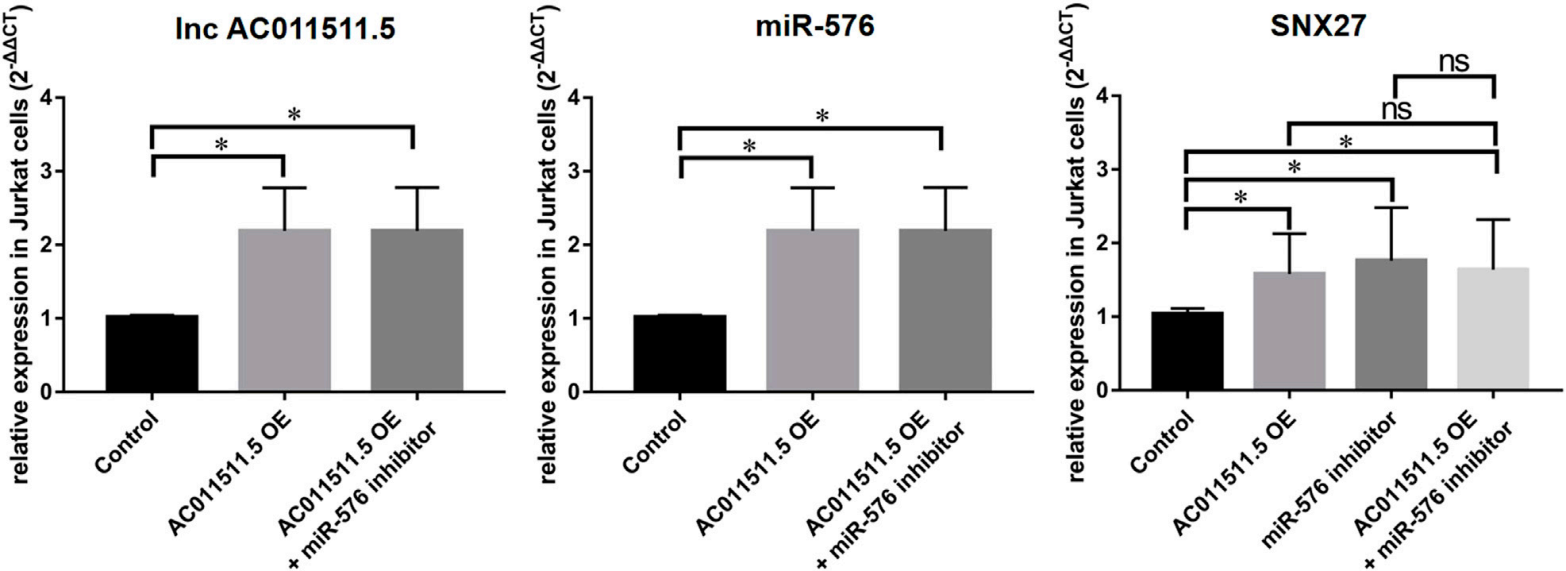
LncRNA AC011511.5 regulates the expression of SNX27 via miR-576-5p in Jurkat cells. *p < 0.05; OE, overexpression.

To explore the regulatory effect of lncRNA AC011511.5/miR-576-5p/SNX27 axis, lncRNA AC011511.5 overexpressing lentivirus or miR-576-5p shRNA contained in lentivirus was transfected into Jurkat cells. We found that overexpression of lncRNA AC011511.5 or inhibitor of miR-576-5p both upregulated SNX27 expression (*p* = 0.016 and 0.014, respectively). Moreover, the levels of SNX27 expression were increased in the miR-576-5p inhibitor & lncRNA AC011511.5 overexpression group (*p* = 0.027). The levels of SNX27 expression in the lncRNA AC011511.5 overexpression and miR-576-5p inhibitor group showed no significant difference compared with miR-576-5p inhibitor group or lncRNA AC011511.5 overexpression group (*p* = 0.648 and 0.816, respectively), indicating that overexpression of lncRNA AC011511.5 could not further upregulate the expression of SNX27 in miR-576-5p inhibitor Jurkat cells (Figure 8).

## Discussion

The complex interactions among diverse RNA species, including protein-coding messenger RNAs and non-coding RNAs such as miRNAs, lncRNAs, circular RNAs and pseudogenes have be reported. These RNAs communicate with each other and coordinate transcription by competitively binding with shared miRNAs (Tay et al., 2014; Thomson et al., 2016). However, the regulatory mechanism of lncRNA-related ceRNA network in AR remains unclear. To further understand the potential molecular mechanism and function of lncRNAs in AR, we used whole blood for microarray analysis and constructed a lncRNA-miRNA-mRNA regulatory network consisting of 3 lncRNAs, 45 miRNAs, and 75 mRNAs by using differential expression analysis, intersection analysis, and correlation analysis. Ultimately, a hub-network was determined based on 1 lncRNA (AC011511.5), 5 miRNAs (hsa-miR-576-5p, hsa-miR-520c-5p, hsa-miR-519b-5p, hsa-miR-519c-5p, and hsa-miR-518d-5p), and 2 mRNAs (ZFP36L1 and SNX27). Following further verification, we found that the lncRNA AC011511.5/miR-576-5p/SNX27 axis might be a key pathway for the pathogenesis of AR.

Although numerous genes are involved in AR, it is unclear how those genes are regulated in the pathogenesis of AR. Recently, thousands of noncoding RNAs such as miRNAs, lncRNAs and cirRNAs have been discovered, and the mechanism of their regulation coding genes have been studied. Studies have shown that lncRNAs can regulate the expression of AR-related genes and participate in the pathological process of AR (Ghafouri-Fard et al., 2020; Zhu et al., 2020). For example, the highly expressed lncGAS5 was found to promote type 2 T helper (Th2) cells differentiation and aggravate the nasal symptoms of AR mice (Zhu et al., 2020). The expression level of lncRNA TCONS_00147848 in the nasal mucosa of AR mice was significantly increased, and it could regulate the apoptosis and inflammation of nasal mucosa cells through the JAK2/STAT3 signaling pathway and participate in the pathogenesis of AR (Huang et al., 2021). There were 18 lncRNAs differentially expressed in the peripheral blood of AR patients when a *p*-value < 0.05 and |Log2Fc| > 1.0 were considered to signify differential expression. At present, there is no report concerning the biological functions and mechanisms of those lncRNAs.

In order to understand their biological functions including GO and KEGG, we conducted a network enrichment analysis on the 75 mRNAs that were co-expressed along with the lncRNAs in the ceRNA. We found that the mRNAs were mainly involved in negative regulation of immune response, response to interferon-beta, and response to interferon-alpha. Type 2 immunity involves the mechanisms by which the immune system responds to worms and a range of environmental substances, such as allergens. Th2 cells play an important role in AR, a systemic immune inflammatory response (Romagnani 2004; Rodriguez et al., 2007; de Kouchkovsky et al., 2017). A previous study showed that impaired interferon (IFN)-related immune responses can lead to increased susceptibility to viral infections, which will lead to certain allergic respiratory diseases (Jeon et al., 2018). The KEGG pathway analysis showed that 75 mRNAs in AR were significantly enriched in NOD-like receptor signaling pathway and tryptophan metabolism, etc. After rhinovirus infection, pyrin domain containing 3 (NLRP3), a member of NOD-like receptor family, contributes to inflammation, pyroptosis, and mucin production in human airway epithelium (Liu et al., 2019). A previous study found that the concentrations of tryptophan and kynurenine in the blood serum of AR patients were higher than that of control subjects, and the concentration is significantly higher in the season out of pollen season, which indicates that serum tryptophan and kynurenine metabolism could serve as a diagnosis biomarker of AR (Ciprandi et al., 2010). The indicated interactions between the relevant mRNAs were made by PPI network, and there were 4 hub-mRNAs in the PPI, including IFITM2, IFITM3, BST2 and ADAR. Human interferon-induced transmembrane proteins (IFITMs) are proteins that can be induced by IFN and can play an important role in innate antiviral and adaptive immunity (Yao and Yan, 2020). The expression of BST2 was increased after IFNγ treatment and it is a biological ligand for ILT7, which can regulate innate immune (Mahauad-Fernandez and Okeoma, 2015). Previous studies also found that mutations in ADAR (Adenosine deaminase acting on RNA) can cause human immune diseases (Maurano et al, 2021). Those findings suggest that lncRNAs might regulate the expression of target mRNAs and thereby play an important role in AR. Therefore, it is important to further explore the mechanism of interaction between DElncRNAs and DEmRNAs.

Currently, more attention is being given to investigate the underlying function and mechanism of lncRNAs by establishing ceRNA networks (Abdollahzadeh et al., 2019). MiRNAs can silence target mRNAs by facilitating their degradation or inhibiting their translation, and thereby play a pivotal role in AR. MiRNAs can control the development and activation of T and B cells, and regulate immune function and inflammation (Tubita et al., 2021). Abnormally expressed miRNAs may regulate the expression of certain genes and alter specific cellular responses, thereby promoting or leading to the development of AR (Weidner et al., 2021). For example, the expression of miR-375 was decreased in the nasal mucosal epithelium of mice with AR and TNF-α-stimulated nasal mucosa cells and the expression of JAK2 increased. MiR-375 inhibits the apoptosis of nasal mucosal cells and improves AR by inhibiting the JAK2/STAT3 pathway (Wang et al., 2018). miR-556-5p targets both the 3′ untranslated region (3′UTR) of NLRP3 and hsa-circ-0000520. mir-556-5p inhibits the expression of has-circ-0000520 in a ceRNA-dependent manner and affects NLRP3-mediated inactive epithelial pyroptosis. The has-circ-0000520/miR-556-5p/NLRP3 signaling pathway was reported to be a possible novel therapeutic target for AR progression (Yu et al., 2021). Our previous research also found that miR-146a can enhance regulatory T-cell differentiation and function in AR by targeting STAT5b (Zhang et al., 2021).

In order to seek candidate genes and explore the novel mechanisms of lncRNAs, we constructed a hub-ceRNA consisting of lncRNA AC011511.5, 5 miRNAs (hsa-miR-576-5p, hsa-miR-520c-5p, hsa-miR-519b-5p, hsa-miR-519c-5p, and hsa-miR-518d-5p), and 2 mRNAs (SNX27 and ZFP36L1). ZFP36L1 is a member of a small family of RNA-binding proteins composed by ZFP36 (also known as tristetraprolin, TTP), which can bind to adenine uridine-rich element (ARE) in the 3’untranslated region of target messenger RNA and stimulate target degradation (Makita et al., 2020). SNX27 is a member of the sorting nexins (SNX) protein family, which helps to regulate intracellular protein trafficking and endosomal signaling (Ghai et al., 2015). In AR, allergen exposure and sensitization involve immune cells such as antigen-presenting cells, T and B lymphocytes, and these immune cells are mainly in PBMCs (Eifan and Durham, 2016). In order to evaluate the regulatory effect of lncRNAs on these immune cells more accurately, we performed validation with PBMCs. Our data indicated that lncRNAs AC011511.5 and SNX27 were expressed at higher levels in patients with AR than in the control subjects, while has-miR-576-5p was expressed at lower levels in the AR patients than in the control subjects. We also found that lncRNA AC011511.5 could act upstream of miR-576-5p to regulate the expression of SNX27. Those findings suggest that a lncRNA AC011511.5/miR-576-5p/SNX27 axis may be a key pathway in AR. However, although SNX27 plays an important role in the pathological process of many diseases, the mechanism of SNX27 in AR needs to be further studied.

## Conclusion

In summary, we constructed a novel lncRNA-miRNA-mRNA network related to AR and verified the regulatory relationship between them. The network provides new insights to explore the molecular mechanism of AR and identify new biomarkers for AR. Our findings improve the current understanding of role of RNAs in the ceRNA in the pathogenesis of AR. In the next phase of our research, we will further explore the function of lncRNA AC011511.5/miR-576-5p/SNX27 pathway in AR.

## Data Availability

The datasets presented in this study can be found in online repositories. The names of the repository/repositories and accession number(s) can be found in the article/Supplementary Material.
